# Temperature dependence of molybdenum dialkyl dithiocarbamate (MoDTC) tribofilms via time-resolved Raman spectroscopy

**DOI:** 10.1038/s41598-021-81326-0

**Published:** 2021-02-11

**Authors:** Carlos E. Garcia, Mao Ueda, Hugh Spikes, Janet S. S. Wong

**Affiliations:** Department of Mechanical Engineering, Imperial College London, London, SW7 2AZ UK

**Keywords:** Engineering, Mechanical engineering

## Abstract

Molybdenum dialkyl dithiocarbamate (MoDTC) is a friction reducing additive commonly used in lubricants. MoDTC works by forming a low-friction molybdenum disulphide (MoS_2_) film (tribofilm) on rubbed surfaces. MoDTC-induced MoS_2_ tribofilms have been studied extensively *ex-situ*; however, there is no consensus on the chemical mechanism of its formation process. By combining Raman spectroscopy with a tribometer, effects of temperature and shear stress on MoS_2_ tribofilm formation in steel-steel contacts were examined. Time-resolved Raman spectra of the tribofilm were acquired*,* together with the instantaneous friction coefficient. The tribofilm is constantly being formed and removed mechanically during rubbing. Increasing shear stress promotes MoS_2_ formation. The nature of the tribofilm is temperature-dependent, with high-temperature tribofilms giving a higher friction than lower temperature films. Below a critical temperature *T*_c_, a small amount of MoS_2_ gives significant friction reduction. Above *T*_c,_ a patchy film with more MoS_2_, together with a substantial amount of amorphous carbon attributed to base oil degradation, forms. The composition of this tribofilm evolves during rubbing and a temporal correlation is found between carbon signal intensity and friction. Our results highlight the mechanochemical nature of tribofilm formation process and the role of oil degradation in the effectiveness of friction modifier MoDTC.

## Introduction

Around 20% of the energy consumed every year is lost to friction in mechanical contacts^1^. Reductions in friction translate into significant improvements in energy efficiency, particularly in transport and power generation^1^. Low viscosity oils are increasingly used^2,3^ because they can reduce frictional losses. They are expected to perform under wide ranges of temperature, speed and load. For this reason, a plethora of oil additives is employed in commercial formulations, including friction modifiers, the focus of this study.

Molybdenum dialkyl dithiocarbamate (MoDTC) is a friction modifier lubricant additive widely used alone or in combination with anti-wear additives such as zinc dialkyldithiophosphates (ZDDP). It has been the subject of extensive research since the 1970s. The friction reduction properties of MoDTC are known to be a consequence of the formation of molybdenum disulphide (MoS_2_) flakes at the asperity peaks of rubbing surfaces, with a size of about 10–30 nm in diameter and a thickness of just a few atomic layers. These flakes result in a patchy-albeit effective-surface layer, often called a tribofilm, that can reduce the friction coefficient from typical values of 0.1–0.12 to as low as 0.05–0.08^4^. MoS_2_ has a layered structure. Friction anisotropy during interlayer sliding, stemming from MoS_2_ lattice incommensurability, is shown to be responsible for friction reduction in a MoS_2_ film^5–8^. Some authors have found correlations between MoS_2_ surface coverage and macroscopic friction^9,10^. Recent studies have also shown that a constant supply of MoDTC is needed to maintain friction low; if the contact is starved of the additive, the MoS_2_ film eventually disappears^11^.

The chemical pathways by which MoDTC decomposes upon rubbing and the subsequent formation of MoS_2_ remain contentious. Several models can be found in the literature. Grossiord et al. introduced a mechanism by which MoDTC breaks up into two free radicals, one of which recombines to form thiuram disulphide and the other one decomposes into molybdenum oxides and MoS_2_; however, no considerations are made about the role of shear stress in the process^12^. De Barros Bouchet et al. modified this model to explain MoS_2_ formation in the presence of zinc dithiophosphate (ZDTP), suggesting that ZDTP acts as a net donor of sulfur atoms^13^. Khaemba et al. proposed a two-step mechanism: first, MoDTC on the surface is subject to shear stress and produces MoO_x_ and iron oxides. Later, MoO_x_ is converted to MoS_2_ and FeMoO_4_ is formed from iron oxides^14^. More recently, Kurihara and Phan^15^ presented a five-step mechanism where MoS_2_ starts forming immediately as a result of the interaction between sulfur atoms in MoDTC and iron. Simultaneously, MoDTC also reacts with FeS, which is produced in the first step, to form more MoS_2_, together with other products including thiuram disulphide and ferric molybdate Fe_2_(MoO_4_)_3_. Since MoS_2_ formation is enhanced by an intermediate species, this mechanism may collectively be regarded as an autocatalytic reaction, as originally proposed by Graham et al.^10^.

While film formation has been shown to be a tribochemical process, MoDTC tribofilm removal processes have not received much attention in literature. For example, Xu et al. studied the loss of friction reduction capability as a result of additive starvation^11^. However, removal processes could not simply be ascribed to additive starvation conditions because they are a consequence of rubbing and occur continuously.

In this paper, we set out to explore the kinetics of MoDTC tribofilm formation and removal based on MoS_2_ signal intensity measured by in situ time-resolved Raman spectroscopy, which we correlate to macroscopic friction^16,17^. By examining the formation of MoS_2_ in real time, we investigate:

1. How test temperature affects the effectiveness of MoDTC friction modifier
2. How test temperature affects the formation and nature of the MoS_2_ tribofilm

## Methods

### Tribometer

Tribological tests were performed using an MTM2 ball-on-disc tribometer from PCS Instruments. In this device, a ball and the flat surface of a disc are pressed against each other to form a contact. The ball and the disc are rotated by independent motors, allowing different disc and ball surface speeds (${U}_{b}$ and ${U}_{d}$ respectively) at the contact to be established. A cantilever loading system applies the required force on the shaft pushing the ball against the disc, while the resulting frictional force is measured by a load cell attached laterally to the shaft at an acquisition rate of 1 Hz. During a friction test, a circular contact track is formed on the disc. The friction coefficient is calculated and its variation with time is recorded to produce a friction curve. Friction curves shown in this work are the result of applying a FFT smoothing filter to the friction signal with a cutoff frequency of 0.33 Hz, hence supressing high-frequency fluctuations.

All specimens (see Table 1) were sourced from PCS and were from the same manufactured batch. They were cleaned prior to test by submerging in a toluene ultrasonic bath for 30 min, followed by a further ultrasonic bath in acetone. Any remaining residue was removed using a hexane-soaked lint free wipe. The specimen was then submerged without delay in the testing oil blend to minimise oxidation or contamination of the surface.

**Table 1:** Dimensions and materials properties of rubbing surfaces.

	Ball	Disc
Dimensions	19.05 mm diameter	46 mm disc diameter, 42 mm track diameter, 5 mm thickness
Materials	AISI 52,100 steel	AISI 52,100 steel
Elastic Modulus (GPa)	207	207
Hardness (Hv)	850 Hv	835 Hv
Poisson ratio	0.293	0.293
Surface Roughness (nm)	< 10 nm	< 10 nm

### Raman spectroscopy

The surface of the disc was examined by Raman spectroscopy. A monochromatic laser beam is focussed on a small spot on the sample surface and the scattered light is collected. The elastic component of the scattered light is optically filtered out and the inelastically scattered light is analysed in a spectrometer. The spectral signature of a substance is determined by the transitions between molecular vibrational states. The Raman spectrum of the MoDTC additive is complex due to its many vibrational modes possible. In the case of MoS_2_, however, there are two Raman-active transitions corresponding to the in-plane and out-of-plane vibrations of the molecule, E_2g_^1^ and A_1g_, that result in strong spectral peaks near 380 and 410 cm^−1^. Other species potentially relevant to the chemistry of this system also possess distinct Raman spectral peaks, including MoO_3_ (820 and 994 cm^−1^) and Fe_2_O_3_ (615 cm^−1^). Should the base oil used in this study undergo degradation and form amorphous carbon, peaks near 1350 and 1580 cm^−1^, commonly known as the D- and G- bands, may also be observed^18^. Reference spectra are provided in SI 1 of the Supplementary Information file.

### Experimental apparatus

**Figure 1:**
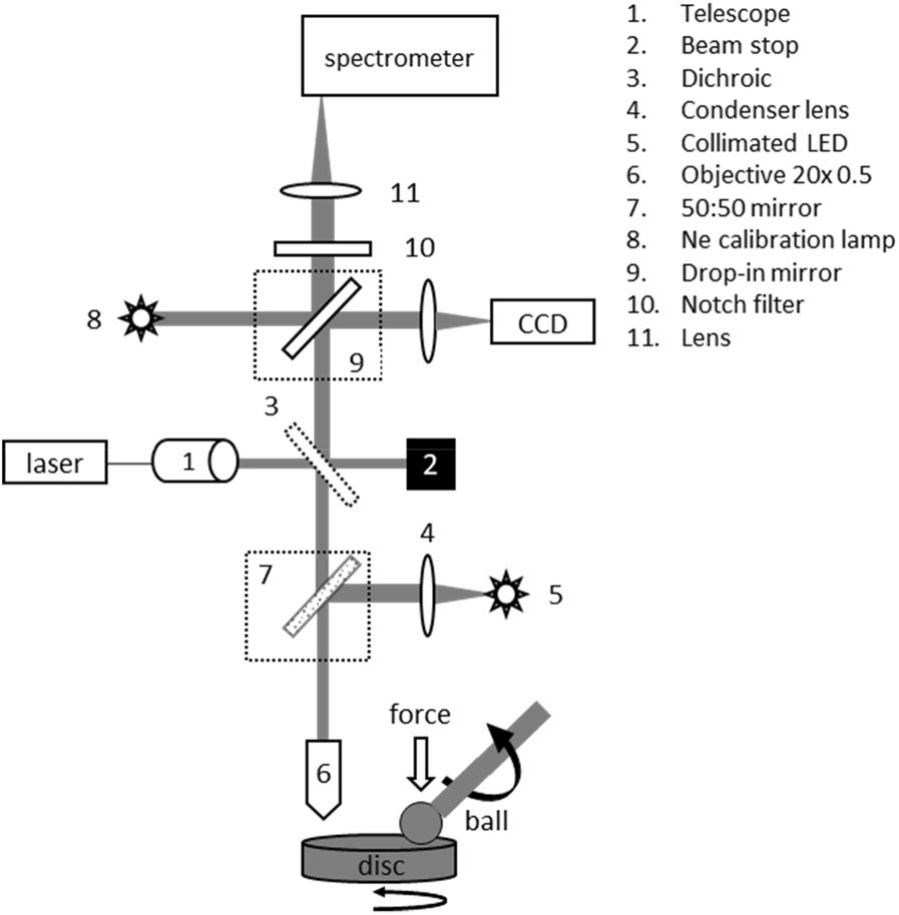
Schematic of the in-situ Raman apparatus.

A custom-built Raman micro-spectrometer was fitted to the tribometer in an epi-illumination configuration, using a 488 nm CW laser (Spectra-Physics Cyan 100 mW), an Andor Shamrock SR-500i spectrometer with an iDus 401 CCD camera, a Falcon EM-CCD camera from Raptor Photonics and a 20 × Olympus microscope objective with NA 0.5, as shown in Fig. 1. The objective approached the surface of the disc vertically, focussing the laser beam on the disc. The laser spot size on the disc was approximately 2 μm. The tribometer was mounted on a 2-axis translatable platform. The position of the tribometer relative to the laser spot was adjusted using micrometric screws. Two additional light sources were built into the apparatus: a visible LED that permitted micrographs of the surface to be taken and a Neon lamp for spectral calibration.

Raman measurements were obtained during rubbing at a location situated 90 degrees upstream of the contact on the disc surface. The friction curve was recorded simultaneously. The Raman signal was collected with an exposure time of 10 s on a continuous cycle and read out immediately (34 ms). The disc undergoes approximately two rotations during each exposure. Hence the resulting spectrum is averaged along the contact track. Raman spectra were collected both at the centre and at the edge of the contact track.

To minimise the amount of oil on the surface at the point of measurement, an air jet was delivered through a 750 µm diameter nozzle to locally push oil away, momentarily exposing the surface as the disc turns. The disc surface becomes fully flooded with oil again before the next pass of the ball occurs. The use of air jet does not affect the formation of the tribofilm (see SI 2 for more information).

### Test conditions

The lubricant contained 1.11 g of a commercial form of MoDTC (Adeka Sakura 165) for every 98.89 g of PAO base oil (Durasyn 164), resulting in a molybdenum concentration of 0.05% wt. To ensure the mixture was well dispersed, it was subjected to a 30-min ultrasonic bath at room temperature prior to each experiment. Unless otherwise stated, tests were performed at a disc and ball speeds of 35 mm/s and 5 mm/s respectively (i.e. entrainment speed of 20 mm/s, a sliding speed of 30 mm/s), and a load of 70 N. This results in a maximum Hertzian contact pressure of 1.24 GPa and a Hertzian contact diameter of approximately 320 µm. Temperature was varied between 60 °C and 110 °C. Based on the test conditions and the properties of the fluid, a Couette-flow EHL calculation^19^ estimated the lubricant film thickness in the contact at 1.7 nm. This is smaller than the surface roughness of contact surfaces. These conditions were chosen so that direct contact between the two rubbing surfaces was expected, a condition that favours MoS_2_ tribofilm formation. All tests had a duration of 1 h. Where MoDTC had been added to the oil, an MoS_2_ tribofilm formed slowly, as evidenced by the rise of MoS_2_ Raman peaks in the collected spectra over time (see Supplementary Information SI 3). A summary of the test conditions is shown in Table 2.

**Table 2:** Range investigated for each experimental test condition.

T (ºC)	Ball speed (mm/s)	Disc speed (mm/s)	Entrainment speed (mm/s)	Force (N)	MoDTC wt% (ppm)	Test duration (min)	Exposure time (s)	Raman location
60–110	5	35	20	30–70	0–500	60	10	Centre–Edge

### Data analysis

**Figure 2:**
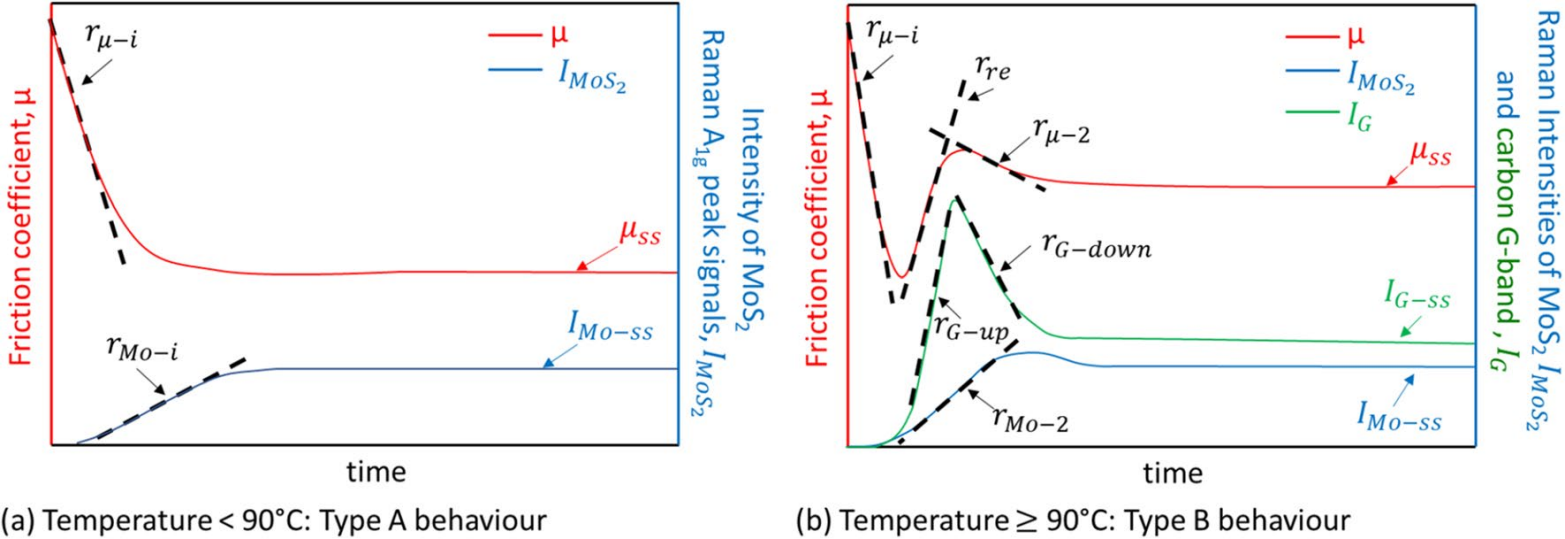
Typical friction curves and time-resolved MoS_2_ and carbon Raman signals indicating how the different rates are defined for test performed at: (**a**) T < 90 °C—type A case; and (**b**) T $\ge$ 90 °C–—type B.

Raman spectra were imported into MATLAB for further processing. A baseline fit was calculated and subtracted from each spectrum. Then, the temporal evolution of the A_1g_ peak intensity, ${I}_{Mo{s}_{2}}$, was computed (schematically shown in blue lines, Fig. 2) and reported with their corresponding friction curves (schematically shown in red lines, Fig. 2).

Depending on test temperature, two types of behaviour were observed. Type A cases occur in tests conducted below 90 °C, where the friction coefficient drops while ${I}_{Mo{s}_{2}}$ rises initially before they both plateau (Fig. 2a). In these cases, the initial rate of increase in ${I}_{Mo{s}_{2}}$, ${r}_{Mo-i}$ was determined from the initial slope of the asymptote fit to the data (blue line, Fig. 2a). Similarly, the initial rate of decrease of the friction coefficient ${r}_{\mu -i}$ was determined from a linear fit of the initial straight portion of the friction curve (red line, Fig. 2a).

Type B cases occur when test temperature $\ge$ 90 °C. In these cases, the friction coefficient rises again after its initial reduction. It then reaches a local maximum before it drops and plateaus (see red line, Fig. 2b). In these cases, two other rates are defined (see red line, Fig. 2b): the rebound rate ${r}_{re}$ is defined as the average rate of increase of friction coefficient between the local minimum and local maximum of the friction curve; and the second rate of decrease of friction coefficient, ${r}_{\mu -2}$, is the average rate of change of friction between the local maximum friction and the steady state friction. When the friction curve exhibits Type B behaviour, a sharp increase in ${I}_{Mo{s}_{2}}$ occurs after the minimum friction coefficient is reached. Hence no ${r}_{Mo-i}$ is estimated, instead ${r}_{Mo-2}$ as shown in Fig. 2b (blue line) is used.

In cases where carbon D-band and G-band are observed in Raman spectra, their intensities, ${I}_{D}$ and ${I}_{G}$ respectively, were monitored. For type A cases, ${r}_{D-i}$ and ${r}_{G-i}$ are defined in the same way as ${r}_{Mo-i}$. For type B cases, their ${I}_{D}$ and ${I}_{G}$ curves show a rise, follows by a drop before reaching a plateau (see green line, Fig. 2b). Their rates of change of intensity,${r}_{D-up}$ and ${r}_{G-up}$, are defined in the same way as ${r}_{re}$; and ${r}_{D-down}$ and ${r}_{G-down}$ the same way as ${r}_{\mu -2}$.

All rates are presented as their absolute value (always positive). Friction coefficient, ${I}_{Mo{S}_{2}}$, and in cases where carbon D- and G- bands are observed, ${I}_{D}$ and ${I}_{G}$, eventually reach steady state values of ${\mu }_{ss}$, ${I}_{Mo-ss}$, ${I}_{D-ss}$ and ${I}_{G-ss}$ respectively. Wherever a single steady state value is provided for friction and Raman signals, it is computed by averaging the steady-state portion of the curve and discarding the initial transients. Since each experimental condition was repeated several times, the error bar represents the standard deviation of the individual measurements.

To distinguish Raman intensities and growth rates taken from spectra obtained at the edge of the contact track from those obtained at the centre of the track, ${()}_{-e}$ are rates and intensities related to edges of tracks.

### Ex-situ surface characterisation

Ex-situ surface characterisation was carried out on contact tracks of steel discs obtained at 60 °C and 110 °C. AFM profiles of contact tracks were acquired using a WiTec alpha300RA with a V-shaped silicon nitride cantilever in contact mode. SEM scans of the surface were obtained using a HITACHI S-3400 N in secondary electrons mode; also, an Oxford Instruments INCA EDX detector was used to measure elemental composition. XPS data was obtained with a ThermoFischer H-Alpha spectrophotometer using a monochromatic Al-K_α_ X-ray source (1486.6 eV) with 200 µm spot size.

## Results

### Rubbing causes the formation of MoS_2_

**Figure 3:**
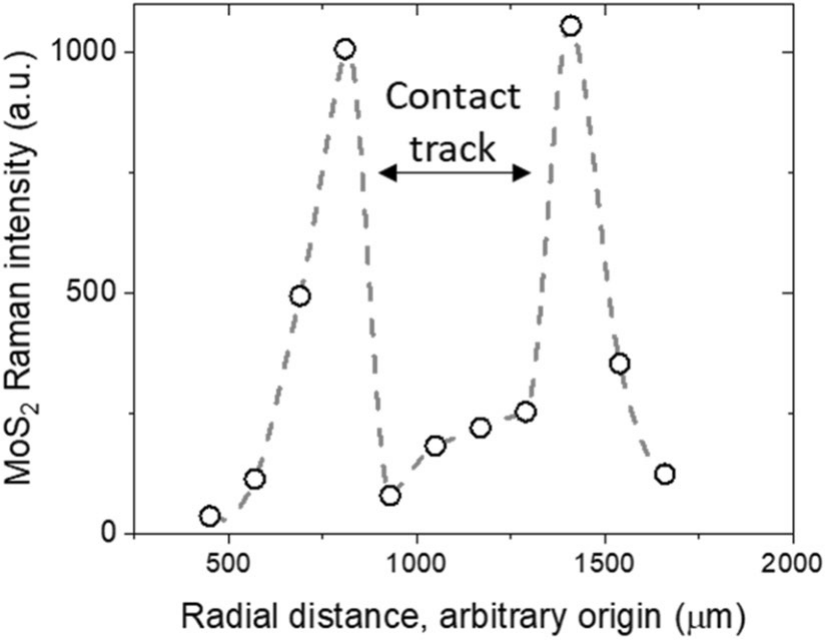
Typical radial MoS_2_ intensity profile across a contact track measured after the test.

The two Raman peaks of MoS_2_, E_2g_^1^ and A_1g_, are detected on contact tracks. In some cases, strong signals from the D (1350 cm^−1^) and G (1580 cm^−1^) bands associated with amorphous carbon also appear (see supplementary information SI 4). A typical ${I}_{Mo{s}_{2}}$ profile across the contact track obtained after a friction experiment is shown in Fig. 3. ${I}_{Mo{s}_{2}}$ at each location is an average of four measurements. MoS_2_ is present at the contact, with a local maximum at the centre of the track observed in some cases. Strong MoS_2_ signals are also detected just outside of the track. This is confined to a region less than one contact diameter ($\sim$ 340 μm) from the track. No MoS_2_ is detected on other regions of the disc surface. The general features of the profiles shown in Fig. 3 are observed in all tests, regardless of whether an air jet was used during the test (see Supplementary Information SI 3).

### Chemical composition of tribofilms based on ex-situ analysis

**Figure 4:**
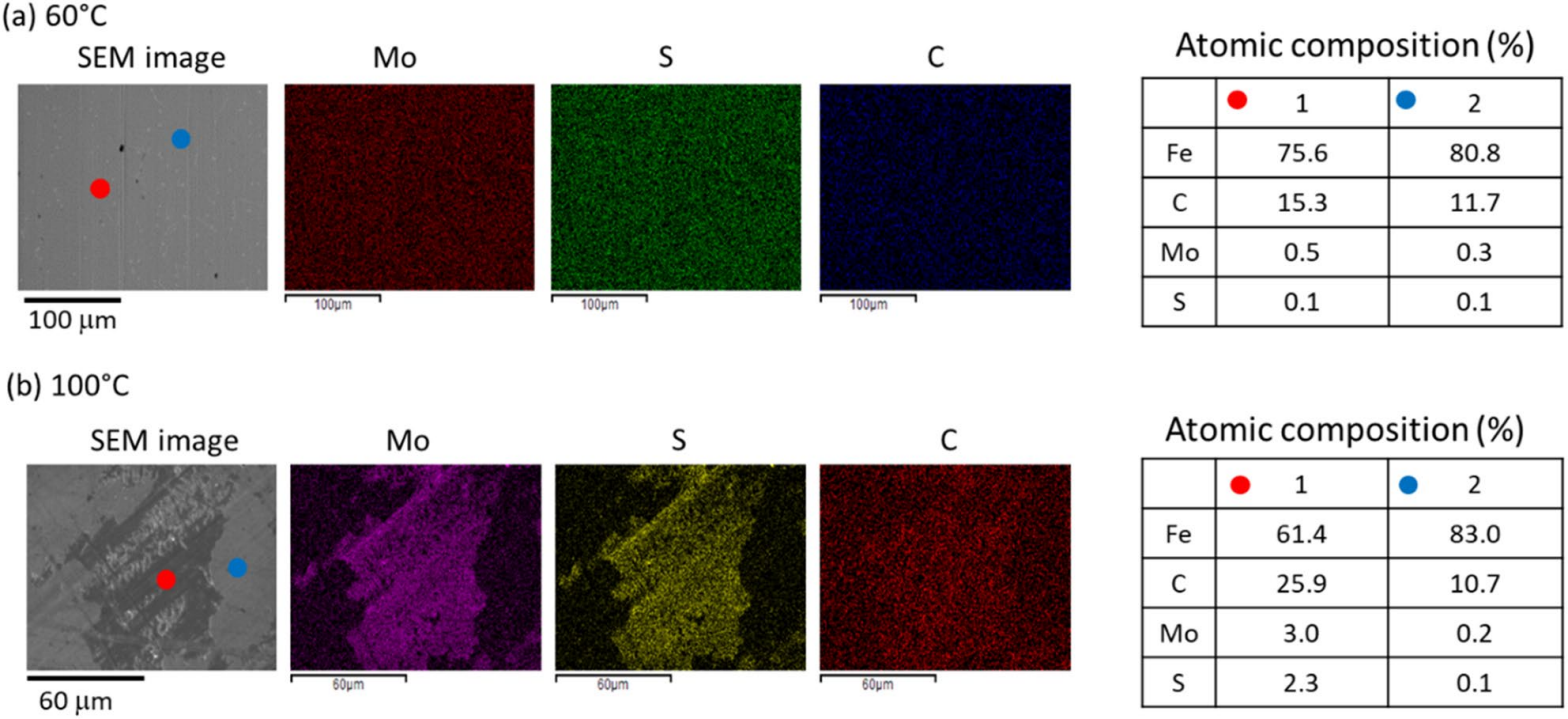
SEM image and EDX scan of contact tracks without wiping showing relative chemical abundance of molybdenum, sulphur and carbon after a test at (**a**) 60 °C and (**b**) 110 °C.

Optical microscopy shows small black patches scattered on the contact tracks (see Fig. S6-1 in SI 6). The amount and size of black patches increase with increasing test temperature. The morphology and the composition of the tribofilms formed at low and high temperatures differ. AFM images of the contact track show a 10–20 nm thick tribofilm after a 60 °C rubbing test (see Fig. S7-2b in SI 7). EDX confirms this thin film contains Mo and S (Fig. 4a). Note that Mo and S are negligible outside the contact track. Thicker patches (> 100 nm, see Fig. S7-1 in SI 7) are found after a 110 °C test. EDX analysis indicates these patches have relatively high Mo and C content (see Fig. 4b). XPS analysis of contact tracks suggest that these patches contain higher fraction of Mo^4+^ than the low temperature tribofilm (see SI 9). These patches can be easily removed with a gentle wipe, after which a 10–20 nm film (Fig. S7-2a in SI 7) of similar Mo and C contents to those of the low-temperature film remains (Fig. S8-1 in SI 8). This implies that the patches adhere weakly to rubbing surfaces. Our results suggest that the high-temperature film is likely to be composed of at least two layers: (1) a top, patchy layer (approximately 100 nm thick), contains relatively high Mo and C contents; and (2) a bottom layer (about tens of nm), with lower C and Mo content, similar to that formed at lower temperature.

### Linking friction reduction to nature of tribofilm

**Figure 5:**
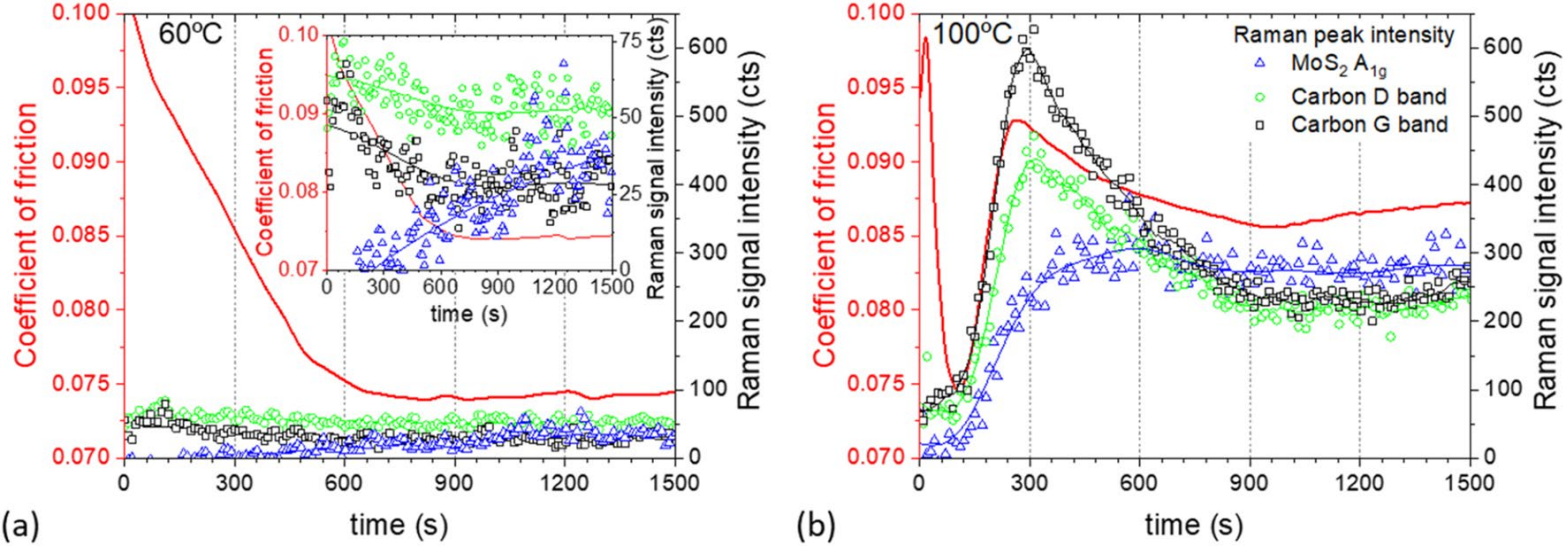
Friction curves and Raman signals measured at the centre of the contact track at (**a**) 60 °C; and (**b**) 100 °C. Data obtained at an applied load of 70 N, disc speed = 35 mm/s and ball speed = 5 mm/s.

Tests conducted at temperatures below 90 °C exhibit Type A behaviour; the friction coefficient gradually reduces until a steady state is reached (red line, Fig. 5a). At the centre of the contact, an increase of ${I}_{Mo{s}_{2}}$ (blue triangles, Fig. 5a) is observed during the test. ${I}_{Mo{s}_{2}}$ continues to rise slightly after the friction coefficient stabilises (see inset in Fig. 5a). Note that ${I}_{D}$ and ${I}_{G}$ (green circles and black squares, Fig. 5a) are relatively constant throughout the test.

Tests conducted at 100 °C and 110 °C show type B behaviour (Fig. 5b); a ‘bump’ is seen in the friction curve after the minimum friction is reached (red, Fig. 5b). This also occurs, although less obviously, in some tests conducted at 90 °C. In these high temperature cases, ${I}_{Mo{s}_{2}}$ (blue triangles), ${I}_{D}$ (green circles) and ${I}_{G}$ (black squares) in the contact track, together with the friction coefficient (red line), rise substantially after friction has reached its minimum. At this rising phase, ${I}_{D}$ and ${I}_{G}$ are higher than ${I}_{Mo{s}_{2}}$. ${I}_{D}$, ${I}_{G}$ and the friction coefficient follow a similar trend: all reach maximum at roughly the same time, before they all decrease and then plateau. On the other hand, ${I}_{Mo{s}_{2}}$ reaches a maximum and then stabilises.

**Figure 6:**
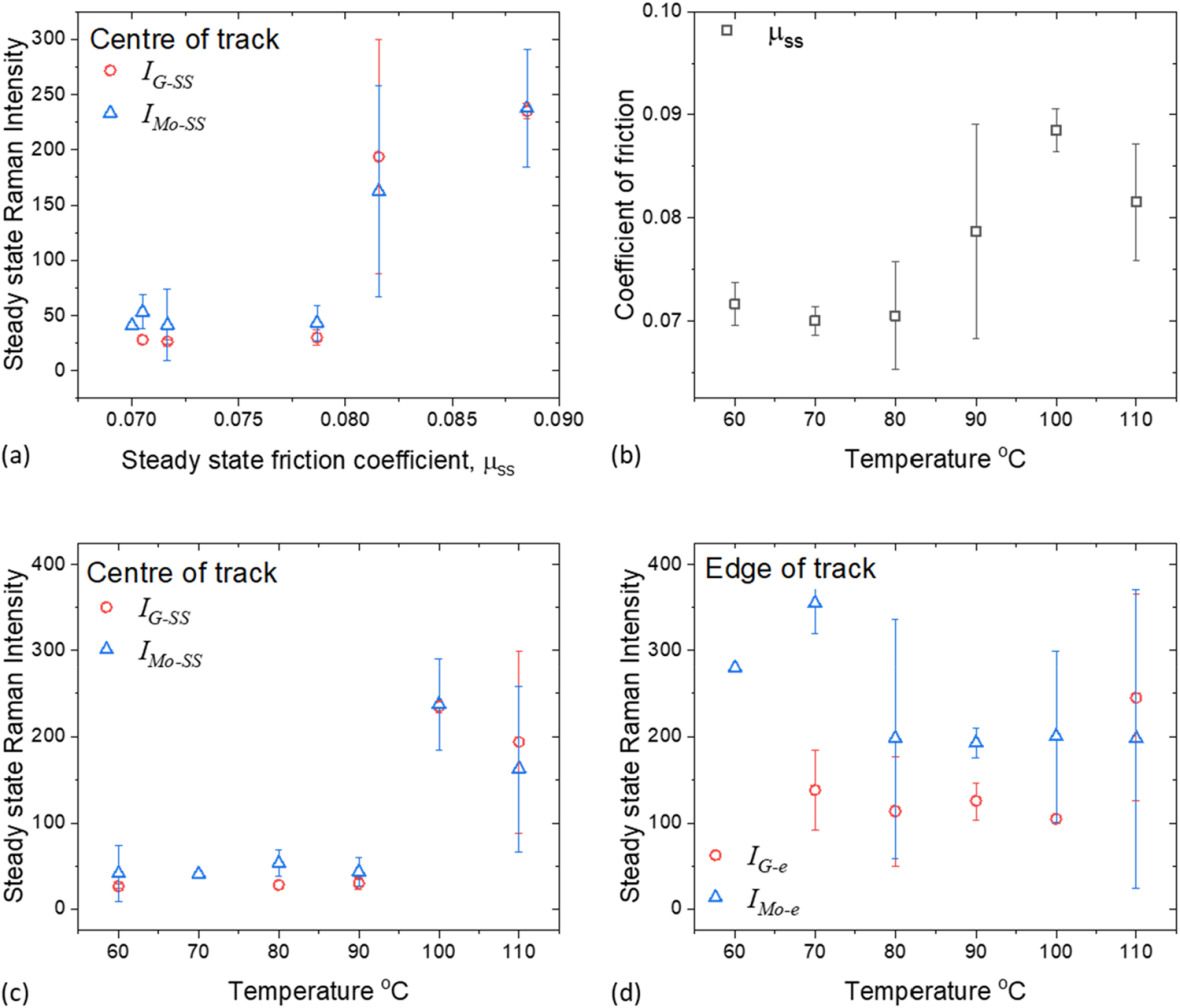
(**a**) The relationship between steady-state Raman intensities of MoS_2_, G-band obtained at the contact track, and steady state friction coefficient, temperature: 60–110 °C and Load = 70 N; the effect of test temperature on (**b**) friction coefficients; (**c**) steady state Raman intensities of MoS_2_, and G-band obtained in the contact track; (**d**) steady state Raman intensities of MoS_2_, and G-band obtained at the edge of the contact track. Note the Raman intensity of D-band is similar to that of G-band in all cases and is omitted for clarity.

The effect of temperature on the steady state friction coefficient and the steady state Raman intensities of MoS_2_, and G-band of carbon obtained in the track are presented in Fig. 6b,c respectively. Note that the Raman intensities of D-band are similar to those of G-band in all cases and are omitted for clarity. At a fixed temperature, ${I}_{Mo-ss}$ (triangles) and ${I}_{G-ss}$ (circles) are similar (see Fig. 6c). ${I}_{Mo-ss}$ obtained at temperatures between 60 and 90 °C have similarly low values, while those from 100 and 110 °C tests are higher. The same is true for ${I}_{D-ss}$ and ${I}_{G-ss}$. This indicates that a transition temperature exists around 90 °C. Such temperature transition is also observed with friction coefficient (Fig. 6b): ${\mu }_{ss}$ is low when test temperature $<$ 90 °C, above which it increases. Our results show that the performance of MoDTC can broadly be divided into two types: low ${I}_{Mo-ss}$, which occurs at low temperature, results in low ${\mu }_{ss}$; while high ${I}_{Mo-ss}$ and high ${\mu }_{ss}$ are observed at higher temperature. The relationship between ${\mu }_{ss}$ and ${I}_{Mo-ss}$ is summarised in Fig. 6a.

### The effect of temperature on friction and MoS_2_ formation

**Figure 7:**
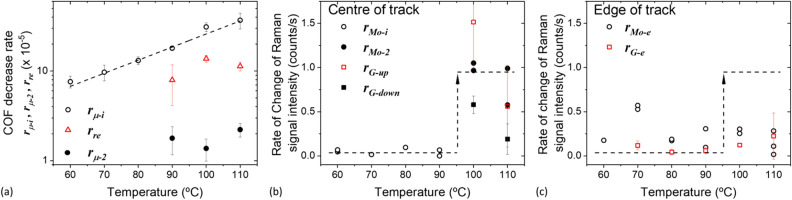
Effect of temperature on: (**a**) rates of change of coefficient of friction (absolute value) and (**b**) rates of change of Raman signals at the centre and (**c**) edge of the track. The dashed lines are for guidance only.

The effect of test temperature on the rates of change of the friction coefficient and various Raman signal intensities are shown in Fig. 7 (for definitions of various rates, see Fig. 2 in ‘Data analysis’ section). The rate of initial decrease in friction coefficients (${r}_{\mu -i}$, open circles, Fig. 7a) appears to follow an exponential increase with temperature. For friction curves which exhibit type B behaviour, friction coefficient increases after it reaches a minimum. The rate of increase in friction coefficient (${r}_{re}$, open triangles) and the rate of its subsequent drop (${r}_{\mu -2}$, solid circles) are estimated (Fig. 7a). Both ${r}_{re}$ and ${r}_{\mu -2}$ are relatively insensitive to test temperature. Note that ${r}_{\mu -2}$ (solid circles) is about an order of magnitude lower than ${r}_{\mu -i}$ (open circles, Fig. 7a).

At low temperature ($\le$ 90 °C), the rate of increase of MoS_2_ intensity (${r}_{Mo-i}$) is low and is temperature-insensitive (${r}_{Mo-i}$, open circles, Fig. 7b). At higher temperatures, ${r}_{Mo-i}$ is not estimated since the period of initial low ${I}_{Mo{s}_{2}}$ is short (see ${I}_{Mo{s}_{2}}$ vs. time in Fig. 5b). This short period of low ${I}_{Mo{s}_{2}}$ is followed by a substantial rise in ${I}_{Mo{s}_{2}}$ and the rate of this delayed ${I}_{Mo{s}_{2}}$ rise is estimated (${r}_{Mo-2}$, solid circles, Fig. 7b). In these high temperature tests, the rate of change in ${I}_{Mo{s}_{2}}$ (${r}_{Mo-2}$, filled circles, Fig. 7b), like the rate of change in friction coefficients (${r}_{re},$ triangle; and ${r}_{\mu -2}$, circles) (Fig. 7a), are insensitive to test temperature. Note that the rate of increase in ${I}_{Mo{s}_{2}}$ from high temperature tests (${r}_{Mo-2}$, filled circles) is higher than that from lower temperature tests (${r}_{Mo-i}$, open circles), see a step change in Fig. 7b. These results confirm a critical temperature around 90 °C exists.

Since strong intensities from carbon D- and G-bands are observed in high temperature tests, the rates of growth of ${I}_{G}$ (${r}_{G-up}$) and its rate of subsequent decrease (${r}_{G-down}$) are also presented (open and solid squares respectively in Fig. 7b). ${r}_{G-up}$ and ${r}_{G-down}$ drop with increasing temperature (open and solid squares respectively, Fig. 7b). It should be noted that tribofilms formed in neat PAO at 110 °C give a very low ${I}_{D}$ and ${I}_{G}$ compared to those from MoDTC–containing PAO in the same test condition (Fig. S5-1 in SI 5).

### The effect of shear on MoS_2_ formation

**Figure 8:**
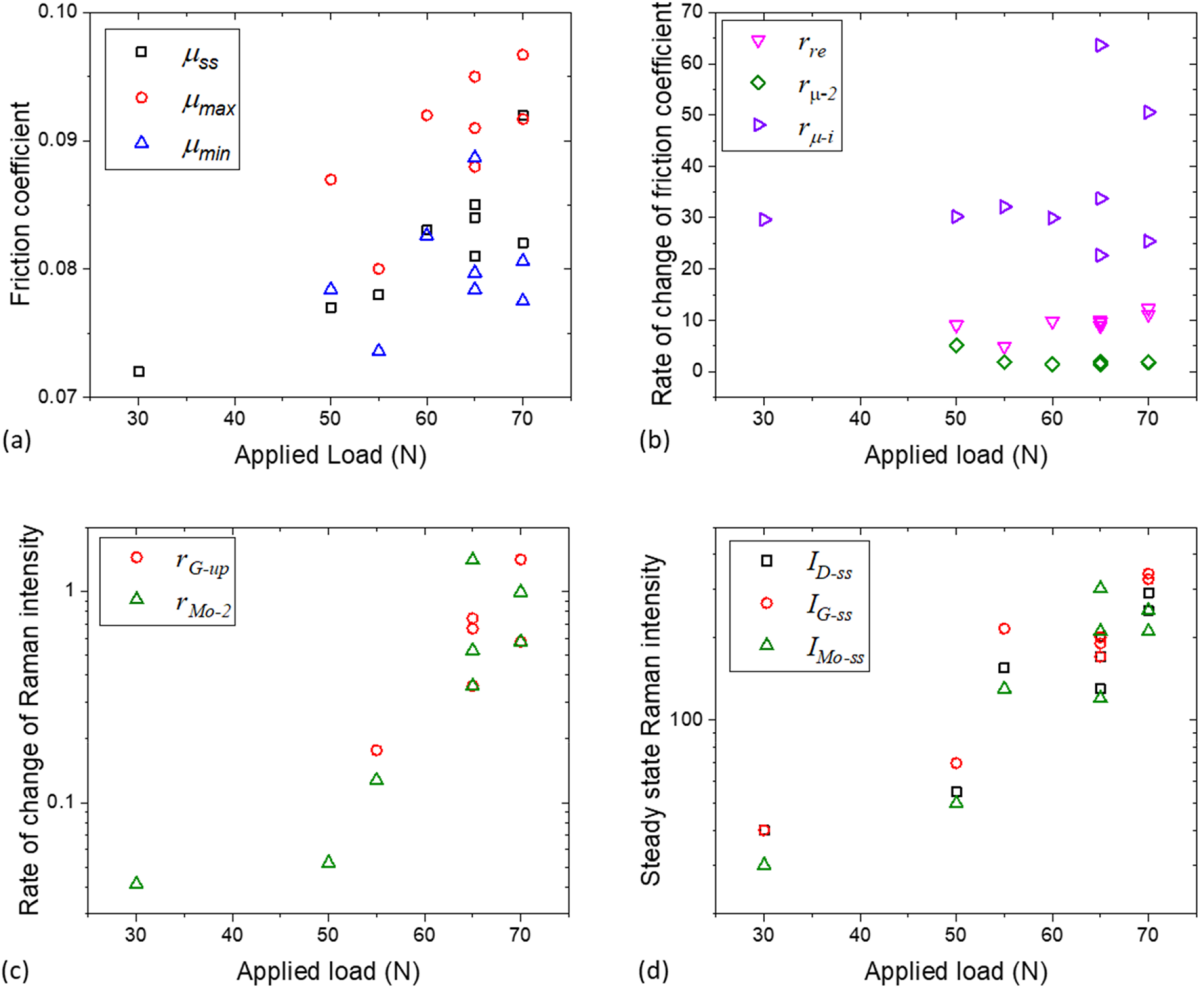
The effect of applied load on (**a**) friction coefficient; (**b**) rate of change of friction coefficient; (**c**) rate of change of intensity of MoS_2_ and carbon G-band Raman peaks; and (**d**) intensity of MoS_2_ and carbon G-band Raman during the steady state. Test temperature = 110 °C.

Experiments were conducted at 110 °C at applied loads ranging from 30 to 70 N. At 30 N, type A behaviour is observed and hence only the steady state friction coefficient is defined. This highlights that both temperature and applied load contribute to the transition from type A to type B behaviour. The minimum ${\mu }_{min}$, maximum ${\mu }_{max}$, and steady state ${\mu }_{ss}$ friction coefficients all increase with increasing load (see Fig. 8a). This effect is the most obvious for ${\mu }_{max}$. Interestingly, the effect of applied load on the rate of change of friction coefficient is weak (Fig. 8b).

**Figure 9:**
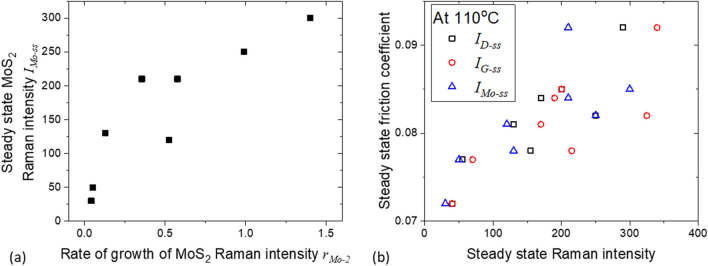
The effect of rate of increase of MoS_2_ peak intensity on (**a**) steady state MoS_2_ intensity and (**b**) steady state friction coefficient. Test temperature = 110 °C, applied load = 30–70 N.

Increasing the applied load promotes the growth of MoS_2_ and carbon, as shown by rising rates of increase of ${I}_{Mo{s}_{2}}$ (${r}_{Mo-2}$, triangles) and of ${I}_{G}$ (${r}_{G-up}$, circles) in Fig. 8c. The rise in these growth rates results in higher Raman intensities of MoS_2_ and D- and G- bands (Fig. 8d, see also Fig. 9a) and higher friction coefficient when the steady state is reached (Fig. 9b). Note that at a fixed load, ${I}_{Mo-ss}$, ${I}_{G-ss}$ and ${I}_{D-ss}$ are comparable (Fig. 8d). This is also seen in tests conducted at lower temperatures (Fig. 6c).

### The accumulation of MoS_2_ outside of contact tracks

**Figure 10:**
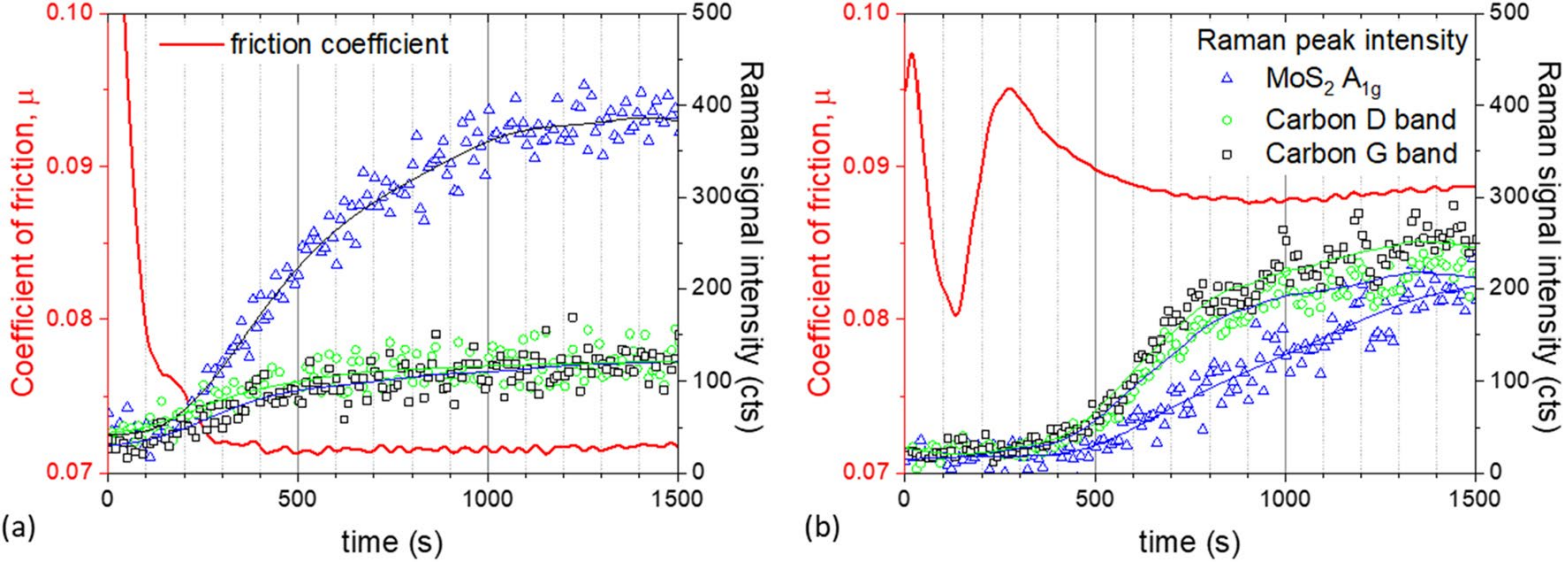
Friction curve and Raman signals measured at the side of the wear track at (**a**) 70 °C; and (**b**) 110 °C.

Raman spectra obtained at the sides of the contact tracks show that ${I}_{Mo{S}_{2}}$ (triangles, Fig. 10) starts to increase rapidly shortly before steady state friction has been reached. At low test temperatures (Fig. 10a), ${I}_{Mo{S}_{2}}$ (triangles) is higher than ${I}_{G}$ (squares) and ${I}_{D}$ (circles) throughout the test. At the end of the test (see Fig. 6d), the intensity from MoS_2_ peak (triangles) is higher than those of G- (squares) and D- (circles) bands of amorphous carbon although the difference decreases as temperature rises. In addition, the intensities of MoS_2_ peak obtained at the side of the contact track (${I}_{Mo-e}$, triangles, Fig. 6d) is higher than that collected within the contact track (${I}_{Mo-ss}$, triangles, in Fig. 6c).

At high test temperatures (Fig. 9b), ${I}_{Mo{S}_{2}}$ (triangles) is comparable to ${I}_{G}$ (squares) and ${I}_{D}$ (circles) during rubbing. ${I}_{Mo-ss}$ (triangles), ${I}_{G-ss}$ (squares) and ${I}_{D-ss}$ (circles) within the contact track (Fig. 6c) and those at the side of the track (${I}_{Mo-e}$, triangles; ${I}_{G-e}$, squares; and ${I}_{D-e}$, circles respectively in Fig. 6d) are similar. This is very different from what is observed in lower temperature tests where the accumulations produced contain a much higher fraction of MoS_2_.

Focusing on the growth rate of carbon and MoS_2_, the rate of increase in MoS_2_ signals (${r}_{Mo-e}$, circles in Fig. 7c) is slightly higher than that from amorphous carbon (${r}_{G-e}$, squares in Fig. 7c) except at 110 °C and they remain relatively constant with test temperatures. Comparing the growth rate of MoS_2_ in and at the edge of the contact track, the former (${r}_{Mo-i}$, Fig. 7b) is slightly slower than the latter (${r}_{Mo-e}$, Fig. 7c) for tests done $\le$ 90 °C, while ${r}_{Mo-e}$ is lower than ${r}_{Mo-i}$ for tests done $>$ 90 °C.

## Discussion

At a fixed test condition, an increase in MoS_2_ Raman signal intensity over time may indicate either an increase in film thickness or an increase in MoS_2_ surface coverage. In our low-temperature experiments (≤ 90 °C), a small initial increase in ${I}_{Mo{s}_{2}}$ gives a substantial reduction in friction whilst further increases in ${I}_{Mo{s}_{2}}$ do not correlate with any further friction reduction, suggesting that surface coverage increases. This is because friction is determined by the shear strength between MoS_2_ layers. It is likely that MoS_2_ forms at the contacting asperities^20^, where its friction reduction action is most efficient, and later redistributes across the contact. It is then removed from the contact track towards its edge, giving rise to the observed constant ${I}_{Mo-ss}$. Note that the link between a reduction of friction and surface coverage of MoS_2_ has been reported in the literature^11,21^.

The observations of higher friction coefficients with increased ${I}_{Mo{s}_{2}}$ at high temperatures are surprising. The shape of friction curves observed at 100 and 110 °C (Fig. 5b) has been observed by other authors^22^ but its origin is unclear. Note that the observed increase in MoS_2_ Raman signal at higher temperature as compared to low temperature film, is due to higher MoS_2_ content, as well as the formation of thicker film. EDX shows our high temperature tribofilm consists of patches (~ 100 nm thick) of higher carbon content formed above a thin layer with lower carbon content (Fig. 4). In the early stages of film formation, which is likely related to the formation of the bottom low C content layer, friction coefficient is low. Time-resolved Raman spectroscopy reveals that carbon forms rapidly and simultaneously to the observed rebound of the friction coefficient. This is likely related to the thicker, high C content patches formed on top of the lower C content layer. MoS_2_ forms more slowly, but when a steady-state MoS_2_ amount is reached, its presence appears to have the effect of slowing down carbon formation, making the carbon signal also settle at a lower, steady-state level. Our results show that the rebound of the coefficient of friction from its local minimum is attributed to the substantial rise in the amount of carbon in the tribofilm which overwhelms the effect of MoS_2_ formed during the period where friction sees a sharp initial fall. Note that the increased carbon content at high temperature may also lead to carbon impurities being incorporated into the MoS_2_ lattice. These impurities, in turn, could impair the friction anisotropy effect, rendering the tribofilm less effective at reducing friction^5^.

Note that olefins are known to dehydrogenate and break up to form a graphitic tribofilm when the surfaces are covered with a catalytic coating (for example MoN_x_–Cu)^23^. Recently this phenomenon has also been reported on uncoated surfaces^24^. The carbonaceous materials observed in the high temperature tribofilm here may come from the degradation of the base oil PAO. The lack of a more catalytic substrate in our case may explain why high carbon Raman signal is only observed in high temperature tests.

Tests with neat PAO (without MoDTC additive) result in high friction and substantial surface damage. An analysis of the tribofilm generated from neat PAO confirmed that amorphous carbon in the patchy layers formed in MoDTC-doped PAO is likely to be originated from PAO (see SI 5). Tribofilms formed in neat PAO at 110 °C give a very low ${I}_{D}$ and ${I}_{G}$ compared to those from MoDTC containing PAO in the same test condition. These results indicate that firstly, a carbon-rich tribofilm, such as one formed in neat PAO, offers minimum, if any, surface protection in our case and leads to high friction. Secondly, the combination of MoDTC or MoS_2_, with high test temperatures and shear, promotes the formation of carbonaceous materials on rubbing surfaces. This suggests MoDTC, MoS_2_ or their intermediate species promotes the degradation of PAO in our test conditions. Thirdly, the increase in the amount of MoS_2_ in the high temperature tribofilm is not sufficient to reduce friction to the level achieved at low temperature.

Taking results from Figs. 8 and 9 together, a high applied load at high temperature promotes the formation of a tribofilm. This is shown by an increase in the rate of formation of MoS_2_ and carbon. While the tribofilm contains a high levels of MoS_2_ and carbon at the steady state, our results suggest that the observed higher friction coefficient is linked to the higher carbon content. It is possible that, as the amount of carbonaceous materials in the tribofilm increases, the rise in friction coefficient, and hence the shear stress, encourages further production of both carbonaceous materials and MoS_2_. As the amount of MoS_2_ in the tribofilm rises however, the friction coefficient drops due to its lower shear strength, which in turn hampers the growth of carbonaceous materials. A steady state is eventually reached on the composition of the tribofilm and the friction coefficient (see Fig. 5b).

The chemistry of the accumulations at the edge of the contact track provides insights into the nature of the tribofilm in the contact track during rubbing. At low test temperatures, these accumulations consist mainly of MoS_2_, its amount increasing with time (Fig. 10a). Some carbon is also found. Both the amount of carbon and MoS_2_ at the edge are higher than those detected in the contact track (Fig. 6 and 6d). Note that the difference between ${I}_{Mo-e}$ and ${I}_{Mo-ss}$ is larger than those between ${I}_{D-e}$ and ${I}_{D-ss}$, and between ${I}_{G-e}$ and ${I}_{G-ss}$. This suggests that while the initial tribofilm contains carbonaceous materials, the tribofilm formed subsequently is mainly composed of MoS_2_. This low shear strength MoS_2_ is then being pushed away constantly during rubbing and accumulated just outside the track. As a result, the amount of tribofilm on the contact track is always low.

At high test temperatures, accumulations at the edge of contact tracks contain MoS_2_, as well as carbonaceous materials (Fig. 10b), and their amount at steady state are similar to those in the contact track (Fig. 6c vs 6d). This suggests that both carbon and MoS_2_ are continuously formed in the contact and are removed together during rubbing. Friction results show that this mixed tribofilm is not as effective in reducing friction as the MoS_2_ tribofilm.

**Figure 11:**
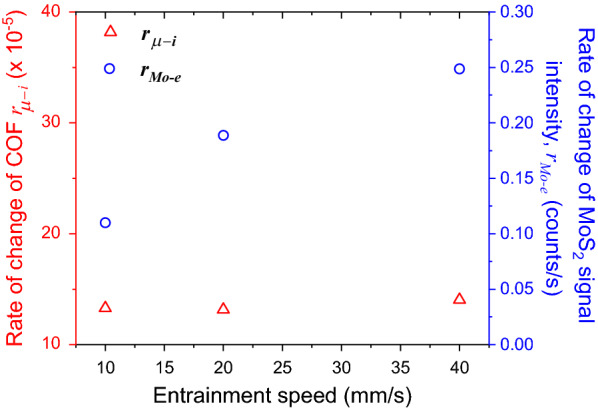
The accumulation rate outside the contact track increases linearly with speed (number of passes), while the rate of change of friction coefficient remains constant. Test conducted at 80 °C, applied load = 70 N and SRR = 150%.

The rate of MoS_2_ accumulation at the edge of the rubbing track (${r}_{Mo-e}$) is temperature independent. Tests conducted at different entrainment speeds show that the rate of MoS_2_ accumulation at the edge of the rubbing track (circles, Fig. 11) increases roughly linearly with entrainment speed, while the rate of change of friction coefficient ${r}_{\mu -i}$ remains constant (triangles, Fig. 11). This suggests that the amount accumulated is governed by the number of contact passes. Our results support that the accumulation process is likely to be mechanically-, rather than chemically-, driven, i.e. MoS_2_ formed in the contact track is pushed to the edge of the track during rubbing (the cross section observed in Fig. 3). Note that the accumulation of MoS_2_ does not affect the rate of friction reduction or the steady state friction coefficient.

**Figure 12:**
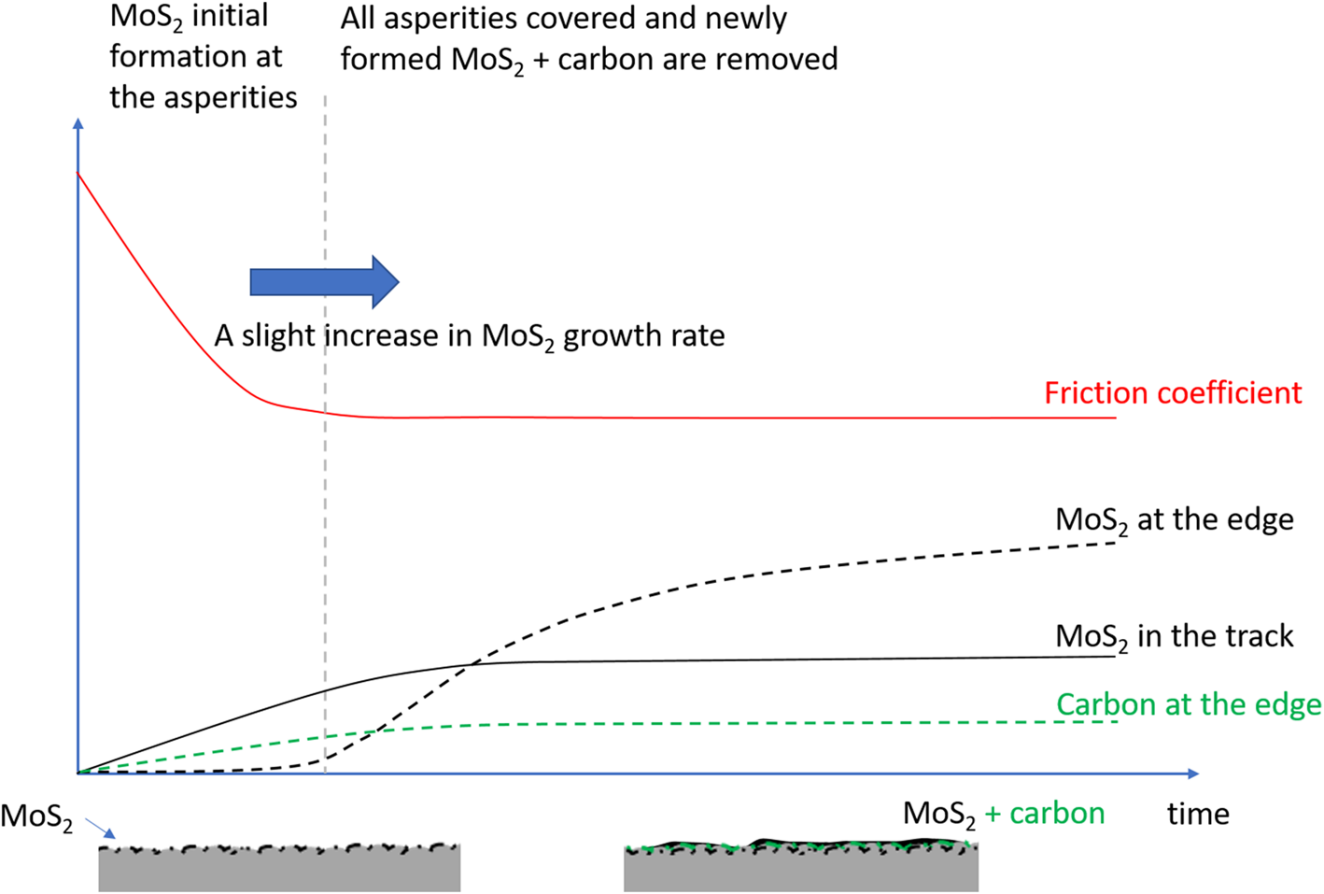
Schematic representation of the variation in friction and Raman signal over time during a low temperature (< 90ºC test).

**Figure 13:**
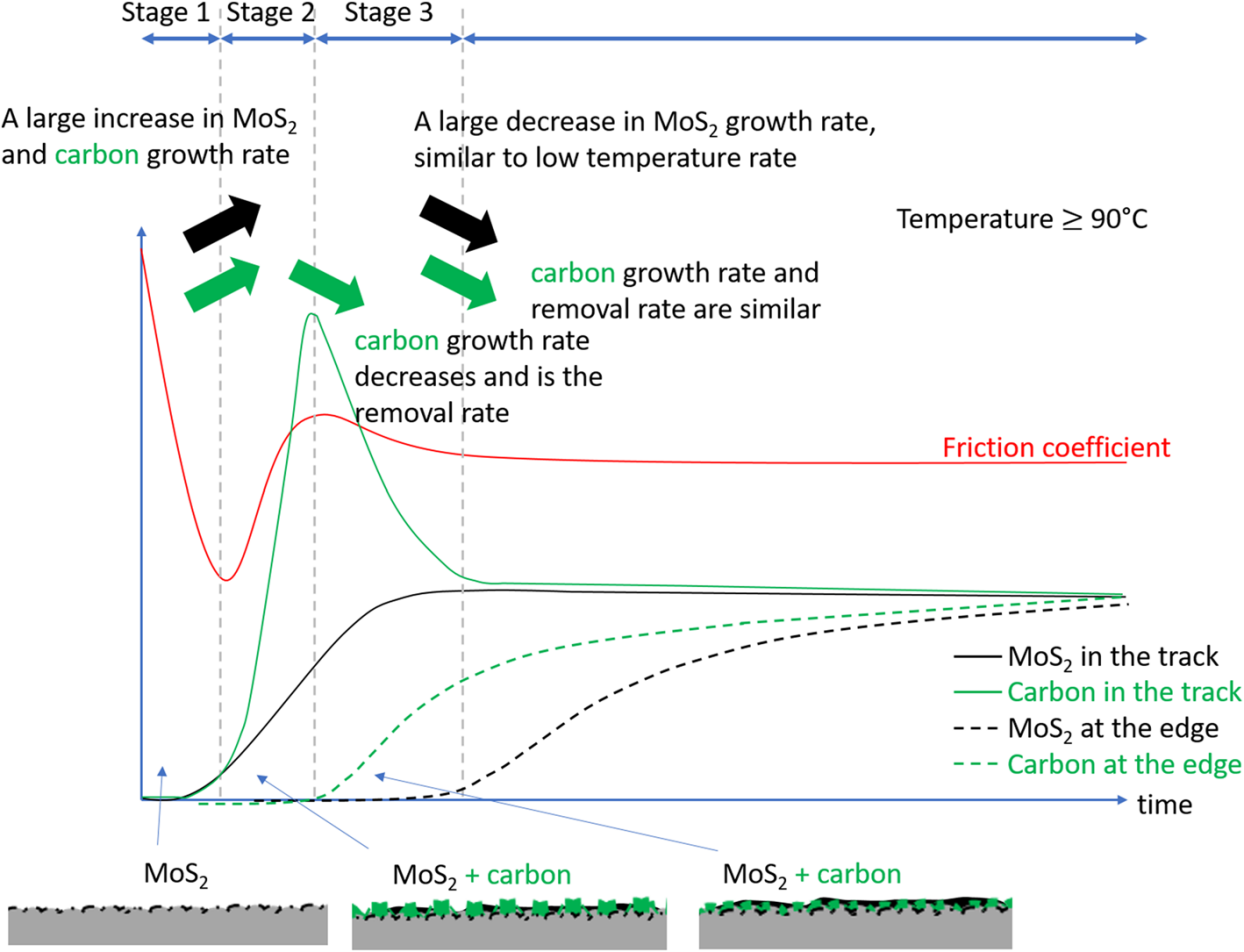
Schematic representation of the variation in friction and Raman signal over time during a high-temperature (> 90ºC test).

Figs. 12 and 13 show schematic representations of the time-resolved Raman signals and friction, interpreted in the context of related literature (shear-induced decomposition of MoDTC is more favourable for the formation of MoS_2_ at asperities, which then gets redistributed around the surface by mechanical processes)^14,17,25,26^. Some features are exaggerated for clarity. At test temperatures $\le$ 90 °C (Fig. 12), the rate of decrease of friction coefficient (${r}_{\mu -i}$) is related to a tribofilm with a small amount of carbon and MoS_2_. Despite its low rate, the growth of MoS_2_ soon overtakes that of carbon. As a result, the accumulation at the edge of the track is MoS_2_-rich. Taken into consideration of tribofilm removal during rubbing, our results suggest the actual growth rates of MoS_2_ initially and during the transition to a stable low friction coefficient are at least equal to the rate of growth of MoS_2_ signals obtained in the track (${r}_{Mo-i}$) and at the edge of the track (${r}_{Mo-e}$) respectively. The small amount of MoS_2_ remained on the contact track is sufficient to provide a low and stable steady state coefficient of friction. This is due to the build-up of MoS_2_ on the contact track^10^, especially at asperities^20^.

At test temperature $\ge$ 90 °C (Fig. 13), the growth of MoS_2_ can be divided into three stages. The initial stage (stage 1) is like that observed $\le$ 90 °C. Although ${r}_{Mo-i}$ values are not estimated in these cases, it is probable that they are similar to those at lower temperature. This is supported by (1) the temperature insensitivity of ${r}_{Mo-i}$ at lower temperature, and (2) the rate of initial drop in friction (${r}_{\mu -1}$) across both temperature ranges can be described by the same exponential relationship. This suggests that the nature of the tribofilms and the friction reduction mechanisms at this stage of the friction curve are likely to be similar across the whole test temperature range.

Stage 2 starts when the friction coefficient is at its minimum. Both the amount of carbon and MoS_2_ increase rapidly. Note that the rate of increase of carbon signals (${r}_{G-up}$) is higher than from MoS_2_ (${r}_{Mo-2}$). Stage 2 ends when friction coefficient reaches a local maximum. At Stage 3, the amount of carbon drops at a rate of ${r}_{G-down}$ while friction coefficient reduces gradually at a rate ${r}_{\mu -2}$. The steady state tribofilm in these cases have more carbon and MoS_2_ than their lower temperature counterpart.

Focusing on the growth of MoS_2_, our results suggest the growth rates of MoS_2_ at stage 1, 2 and 3 are at least ${r}_{Mo-i}$, ${r}_{Mo-2}$ and ${r}_{Mo-e}$ respectively. The growth rate of carbon at stage 2 would be at least, ${r}_{G-up}$ while at stage 3, its maximum rate is ${r}_{G-e}$.

The origin of Stage 2, where the amount of carbon and MoS_2_ rise with friction coefficient, is unclear. Our results show that the formation of carbon is favoured at higher temperature and is likely due to the degradation of the base oil PAO. This suggests that the PAO degradation process has a relatively high energy barrier. Such barrier may be overcome during rubbing by an increase of temperature due to frictional heating and mechanical energy in the form of shear. This may explain the load dependence on the growth of ${I}_{D}$ and ${I}_{G}$. As the carbon tribofilm in this case is ineffective in friction reduction, the increased friction may promote further the growth of carbonaceous materials as a test progresses. Note that a similar shear dependence also applies to the growth of MoS_2_. As the amount of MoS_2_ in the tribofilm rises, friction eventually drops and the conditions become less favourable for the formation of carbon, resulting in a reduction of ${I}_{D}$ and ${I}_{G}$. Eventually, the amount of carbon and MoS_2_ stabilise, together with the coefficient of friction.

## Conclusions

MoDTC is one of the most effective friction reduction additives. It is known to form a MoS_2_ tribofilm on steel surfaces under shear conditions. The detailed formation mechanism of this tribofilm, however, remains elusive. In this study, a new experimental platform combining Raman spectroscopy and a sphere-on-disc tribometer is set up. It allows us to monitor in real time the formation of MoS_2_ tribofilms on steel surfaces lubricated with MoDTC-containing PAO.

As the formation of this tribofilm is a tribochemical process, both temperature and shear stress are important factors. We have shown that the nature of the tribofilm is temperature dependent. A critical temperature of 90 °C is identified for our test conditions. Below 90 °C, the tribofilm is mainly MoS_2_, that provides a low friction coefficient. This tribofilm is constantly being formed and then removed mechanically during rubbing. The balance between the formation and removal process results in only a small amount of MoS_2_ at any time covering the track when steady state is achieved. Above the critical temperature, the tribofilm consists of patches ($\sim$ 100 nm thick) of higher carbon and MoS_2_ content formed above a thin layer with carbon and MoS_2_ content similar to those of the low temperature tribofilm. The high temperature tribofilm is again continuously being formed and removed during rubbing. It gives higher friction than its low temperature counterpart despite having a large amount of MoS_2_. The proportion of MoS_2_ and amorphous carbon in the high temperature tribofilm changes throughout the test, and a positive correlation is found between carbon Raman signal intensity and friction coefficient. Our results suggest that carbonaceous material in the tribofilm originates from PAO degradation. This mixed tribofilm starts off richer in carbon and this is accompanied by a sharp rise in friction. Only when the amount of MoS_2_ is sufficiently high does the friction coefficient drop and plateau at an intermediate level.

Apart from temperature, increasing applied load increases the growth rate and the steady state amount of MoS_2_. This highlights the role of mechanical energy in promoting chemical reactions in a rubbing contact and underscores the formation of this tribofilm is an mechanochemical process.

This work shows that while MoDTC can reduce friction in a wide range of temperatures, its effectiveness is temperature and load dependent due to a change of the nature of the tribofilm. Since carbon in tribofilms formed at higher temperature is attributed to the degradation of PAO, this shows that the choice of the base fluid and its potential reactions can be consequential to the effectiveness of additives and the frequency of lubricant change in engineering applications.

## Supplementary Information

Supplementary Information
