# Supplementary material for: Temperature dependence of molybdenum dialkyl dithiocarbamate (MoDTC) tribofilms via time-resolved Raman spectroscopy
Source: Sci Rep. 2021 Feb 11;11:3621. doi: 10.1038/s41598-021-81326-0 (PMC7878910; doi:10.1038/s41598-021-81326-0)
Supplement: Supplementary file 1 — Supplementary Information [file 41598_2021_81326_MOESM1_ESM.pdf]

Supplementary Information for

# Temperature dependence of Molybdenum dialkyl dithiocarbamate (MoDTC) tribofilms via time-resolved Raman spectroscopy

Carlos E. Garcia[1], Mao Ueda[1], Hugh Spikes[1], Janet S. S. Wong[1*]

[1]Department of Mechanical Engineering, Imperial College London, London SW7 2AZ, UK.

*j.wong@imperial.ac.uk

## SI 1: Reference spectra

Typical Raman spectra measured during this work are shown in Figure S1, including a steel disc surface, MoDTC additive (without oil), $MoS_2$ tribofilm and $MoO_3$.

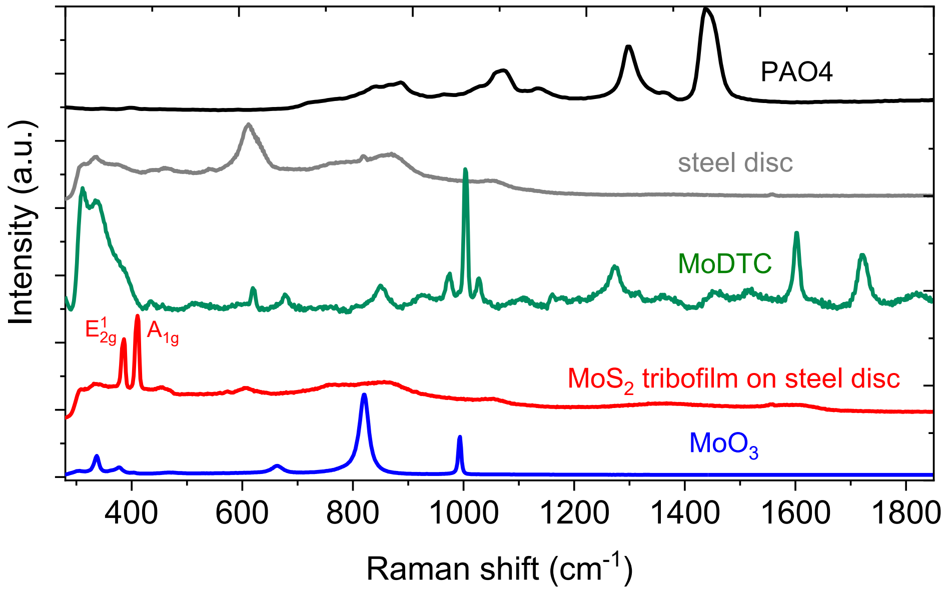


*Figure S1: Typical Raman spectra of (top to bottom): PAO4, steel disc surface, MoDTC, $MoS_2$ and $MoO_3$.*

**SI 2: The effect of air jet**

Typical profiles of the intensity of Raman signal of $MoS_2$ ($I_{Mos_2}$) across the contact track formed under typical test conditions without the use of air jet (red) and after an air jet was blown on the track for 10 mins (blue) (without rubbing) were shown in Figure S2. $I_{Mos_2}$ profile across the contact track formed with the use of air jet can be found in Figure 3 in the main text. They all show similar features. This shows that the use of the air jet has a negligible effect over the $MoS_2$ distribution in the tribofilm.

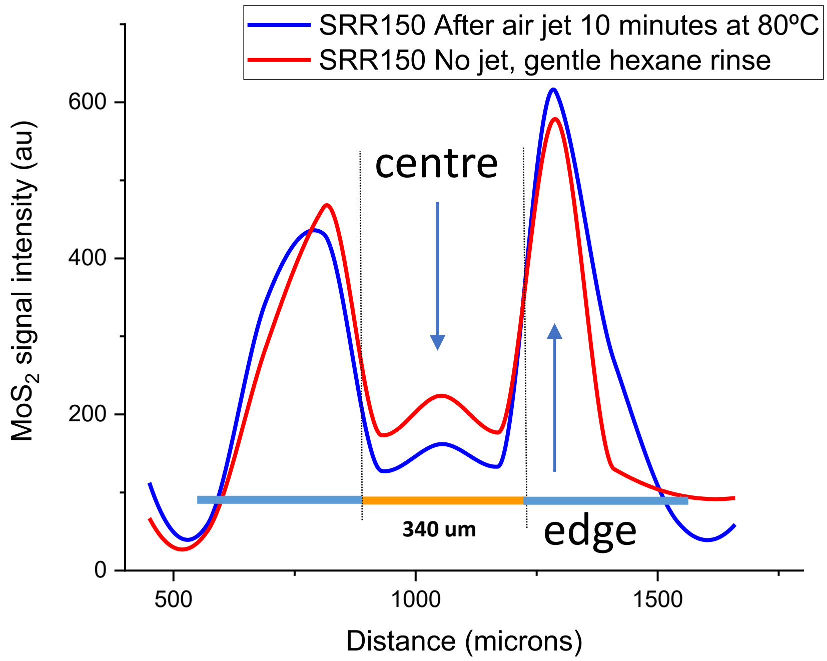


*Figure S2: (a) Radial $I_{Mos_2}$ profiles across contact track, before and after applying air jet. $I_{Mos_2}$ at each location is an average from four measurements.*

$MoS_2$ is present at the contact track, with a local maximum at the centre of the track. An accumulation of $MoS_2$ at the two edges of the track is also detected. Such accumulation is confined to a region less than one Hertzian diameter (~ 340 μm) from the track. No $MoS_2$ can be detected in other parts of the disc.

While some fluctuations in $I_{Mos_2}$ profile are observed between discs, the general features of the profile described above applied to all cases.

**SI 3: examples of evolution of spectra**

Time-resolved Raman spectra were collected in a frequency of 0.1 Hz. When MoDTC has been added to the base oil, the two characteristic $MoS_2$ peaks appear and their intensity progressively increases. Figures S3-1 and S3-2 show the uncorrected Raman spectra at different instants during a rubbing test obtained in the contact track and just outside (edge) the track respectively.

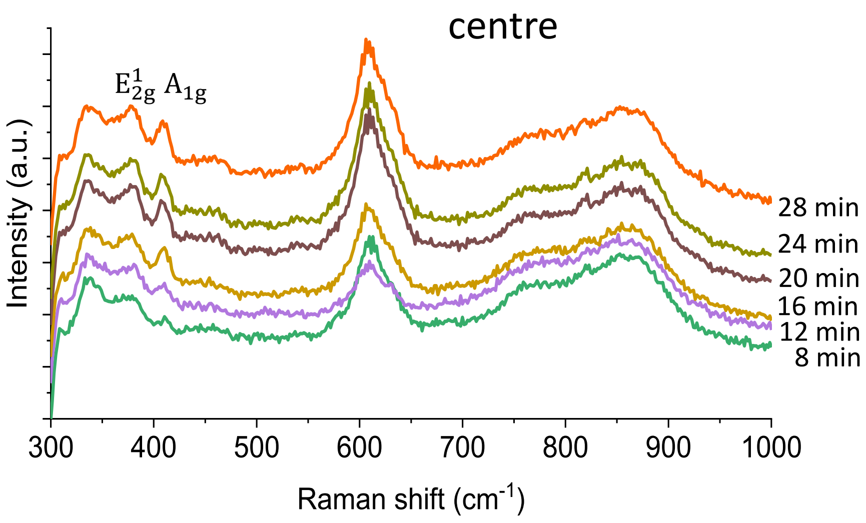


*Figure S3-1 Uncorrected Raman spectra taken in the contact track during rubbing*

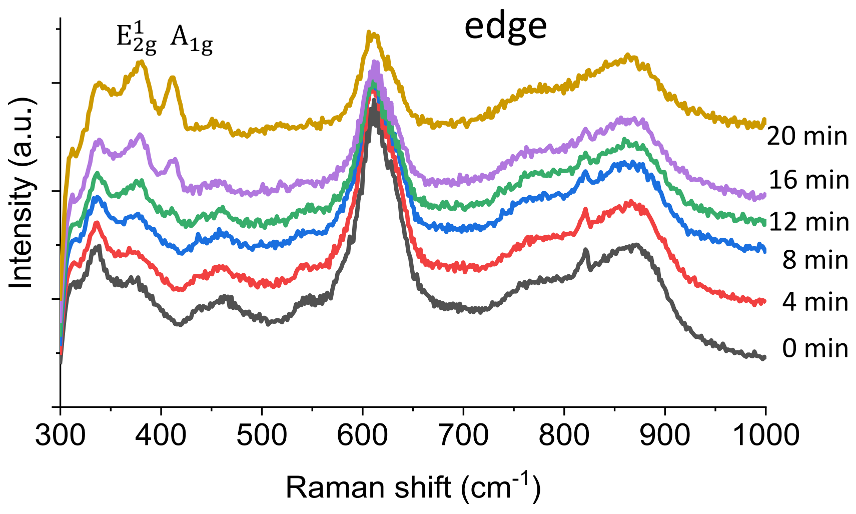


*Figure S3-2 Uncorrected Raman spectra taken just outside the contact track during rubbing*

**SI 4: Comparison of Raman spectra obtained in PAO with and without MoDTC.**

Figure S4 shows spectra on the track after 160 and 350 s rubbing with PAO + MoDTC at 110°C, as well as 3600 s rubbing without MoDTC. These are compared with a spectrum outside the rubbing track. The $MoS_2$ peaks appear after rubbing with PAO + MoDTC. The D- and G- bands appear much sooner when MoDTC is added into PAO.

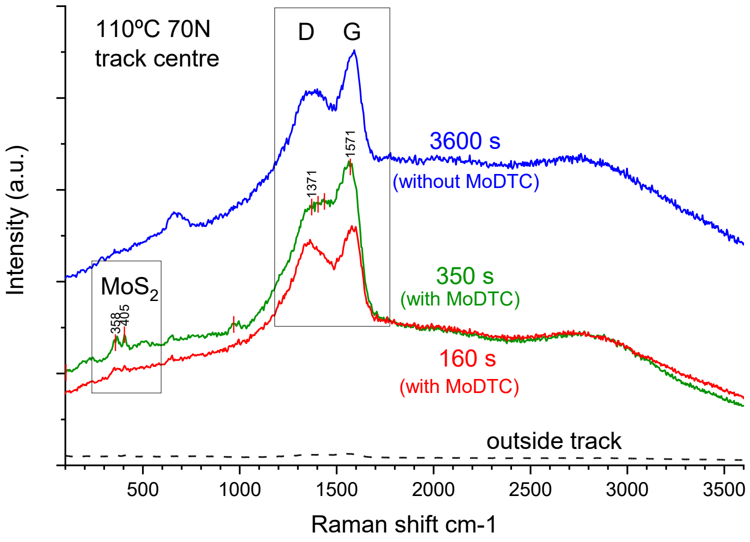


*Figure S4: The raw Raman spectra obtained on the wear track after rubbing in PAO-4 at 110°C with and without MoDTC additive show the D and G bands of amorphous carbon.*

**SI 5: results from neat PAO**

Tests performed with neat PAO (without adding MoDTC) show little or no friction reduction. Friction starts increasing because of surface damage (Figure SR-1).

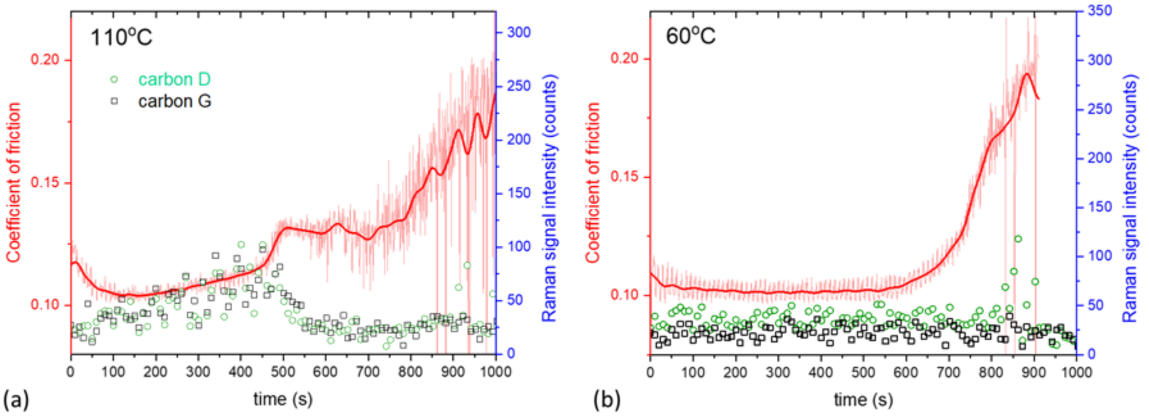


*Figure S5-1: Friction curves with neat PAO: (a) 110ºC and (b) 60ºC. Raman signals were obtained at center of contact tracks*

Another sets of tests with neat PAO were conducted and were stopped after 400 seconds of testing to prevent surface scuffing and mechanical removal of surface films. A carbon-rich film was detected by SEM-EDX on wear tracks formed at 110°C (Figure S5-2), but not at 60°C. Note this carbon rich film has a similar composition to patches formed in PAO+MoDTC at the same temperature. This supports that the source carbon in the tribofilm formed in PAO+MoDTC is degraded PAO.

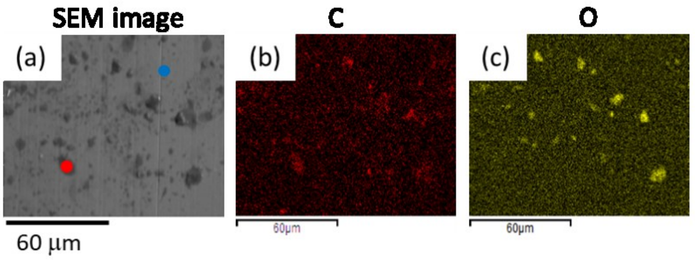


(d) **Atomic composition (%)**

| | ● 1 | ● 2 |
|---|---|---|
| Fe | 64.9 | 82.8 |
| C | 27.6 | 11.5 |
| O | 6.1 | 4.4 |
| Cr | 1.4 | 1.3 |

*Figure S5-2 – SEM image (a) and EDX scan showing relative chemical abundance of: (b) carbon and (c) oxygen after 400 seconds at high temperature (110ºC) with neat PAO (no MoDTC added).*

**SI 6: Optical Images of worn surfaces**

Figure S6-1 presents optical micrographs of contact surfaces obtained at 60 and 110°C.Examining our worn steel surfaces after tests ex-situ by optical microscopy (see Figure S6-1) shows the fraction of the worn surface covered by black substance increases with increasing test temperature. SEM-EDX shows that the worn surface formed at 60°C contains Mo with relatively uniform distribution.

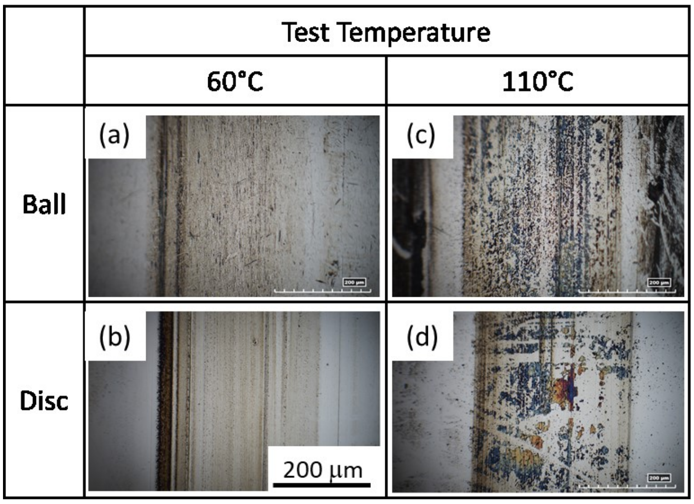


*Figure S6-1 Optical micrographs of worn surfaces after rubbing test before surfaces were wiped.*

**SI 7: AFM images and profile of tribofilm**

At 110°C, AFM image shows thick patches (>100 nm) on the contact track before wiping (Figure S7-1). After wiping, the patches are removed and the underlying profile (Figure S7-2a) is similar to the profile of contact track formed at 60°C (Figure S7-2b).

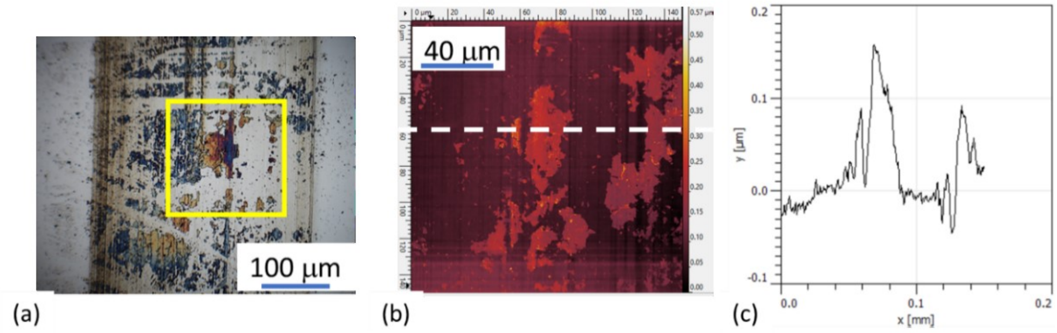


*Figure S7-1 - Surface morphology of a contact track formed at 110°C before wiping: (a) optical image; (b) AFM image of the squared region in (a); and (c) a line profile across the wear track at a local highlighted by the dash line in (b).*

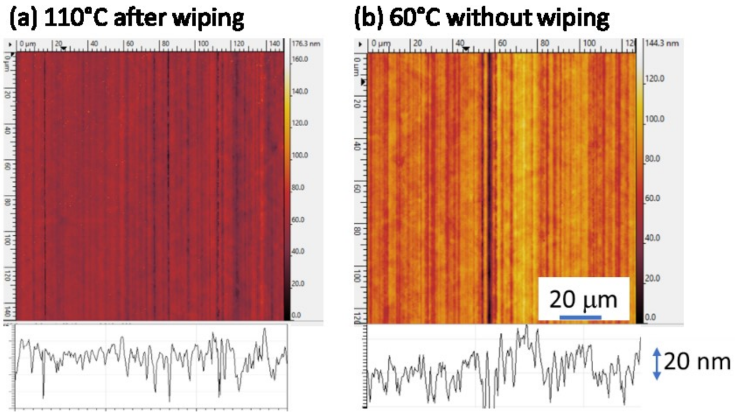


*Figure S7-2 AFM images and profiles of contact tracks formed at (a) 110°C after wiping and (b) 60°C before wiping*

**SI 8: SEM micrograph of contact track formed at 110°C after wiping**

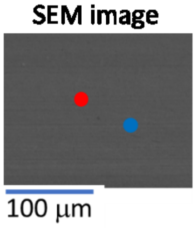


**Atomic composition (%)**

| | 1 (red) | 2 (blue) |
|---|---|---|
| Fe | 81.1 | 81.1 |
| C | 11.1 | 8.5 |
| Mo | 0.5 | 0.7 |
| O | 4.6 | 6.3 |
| S | 0.1 | 0.1 |
| Cr | 1.5 | 1.7 |
| N | 1.1 | 1.6 |

*Figure S8-1 - SEM image and surface composition based on EDX scan of a contact track formed at 110°C after wiping.*

**SI 9: XPS of worn surfaces of steel disc**

XPS of the wear tracks (Figure S9-1) confirms that the tribofilm formed at high temperature contained higher fraction of $Mo^{4+}$ ($MoS_2$) than the low temperature tribofilm. These results support that the anomalous friction curves seen at high temperature stem from a change in chemical composition of the tribofilm. They show, independently from the Raman analysis, that a carbonaceous film is indeed formed at high temperatures but not at low temperatures.

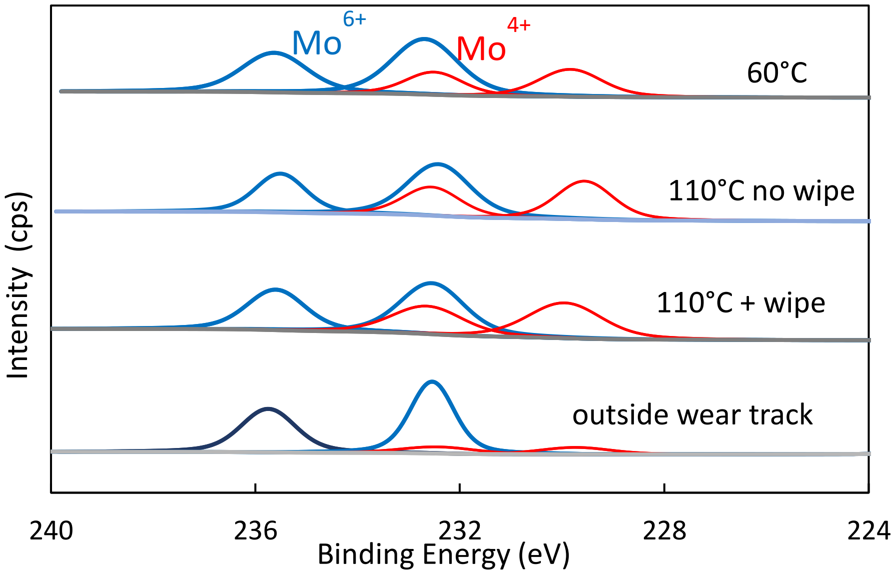


| Condition | $Mo^{4+}$ / ($Mo^{4+}$+$Mo^{6+}$) |
|---|---|
| 60ºC | 0.30 |
| 110ºC no wipe | 0.45 |
| 110ºC + wipe | 0.46 |
| Outside wear track | 0.13 |

*Figure S9-1 – XPS spectra of $Mo^{4+}$ and $Mo^{6+}$ for different conditions and fraction of $Mo^{4+}$*
